# Cardiovascular risk in inflammatory bowel disease: focus on lipids and visceral adipose tissue

**DOI:** 10.3389/fendo.2026.1860937

**Published:** 2026-06-19

**Authors:** Eva Karaskova, David Friedecky, David Kleparnik, Adela Palkovska, Radana Brumarova, David Karasek

**Affiliations:** 1 Department of Pediatrics, University Hospital Olomouc, Faculty of Medicine and Dentistry, Palacky University, Olomouc, Czechia; 2 Laboratory for Inherited Metabolic Disorders, Department of Biochemistry, University Hospital Olomouc, Faculty of Medicine and Dentistry, Palacky University, Olomouc, Czechia; 3 3rd Department of Internal Medicine - Nephrology, Rheumatology and Endocrinology, University Hospital Olomouc, Faculty of Medicine and Dentistry, Palacky University, Olomouc, Czechia

**Keywords:** anti-inflammatory therapy, atherosclerosis, cardiovascular risk, dyslipidemia, gut microbiota, inflammatory bowel disease, lipidomics, lipids

## Abstract

Inflammatory bowel diseases (IBD), including Crohn’s disease and ulcerative colitis, are chronic immune-mediated diseases that are increasingly recognized as systemic diseases with significant cardiovascular consequences. Growing epidemiological evidence suggests that patients with IBD face an increased risk of atherosclerotic cardiovascular disease (ASCVD) that cannot be fully explained by traditional cardiovascular risk factors. This excess risk is most pronounced in younger patients and during periods of active intestinal inflammation. This review summarizes current knowledge on common pathogenic mechanisms linking IBD and ASCVD. Chronic systemic inflammation plays a central role, promoting endothelial dysfunction, hypercoagulability, immune cell activation, and accelerated atherogenesis. Other factors include intestinal barrier disruption with microbial translocation, dysbiosis of the gut microbiome, dysfunctional visceral adipose tissue, and adverse metabolic effects of some IBD therapies. Particular emphasis is placed on lipid abnormalities observed in IBD, including the “lipid paradox”, a phenomenon in which reduced circulating lipid levels paradoxically coexist with increased cardiovascular risk due to inflammation-mediated changes in lipid metabolism leading to lipoprotein dysfunction, and emerging lipidomic biomarkers that suggest causal relationships between specific lipid species, inflammatory mediators, and cardiovascular risk. Attention is also given to current strategies for the assessment and prevention of cardiovascular risk in IBD, emphasizing the importance of controlling disease activity, minimizing corticosteroid exposure, and aggressive treatment of modifiable cardiovascular risk factors. Traditional risk calculators may underestimate risk in this population, highlighting the need for tools that integrate inflammatory burden and imaging of subclinical atherosclerosis. Optimization of anti-inflammatory therapy along with individualized cardiovascular prevention strategies may improve long-term outcomes in patients with IBD.

## 1 Introduction

Inflammatory bowel diseases (IBD), including Crohn’s disease (CD) and ulcerative colitis (UC), are a group of chronic, immune−mediated disorders affecting the gastrointestinal tract. The global prevalence of IBD has increased dramatically over the past three decades, rising from 3.7 million to more than 6.8 million affected individuals (1). The incidence of IBD is increasing worldwide, particularly in regions adopting a “Western lifestyle”. Its key components – such as high consumption of saturated fat, refined carbohydrates and ultra-processed foods – play a central role in both the pathogenesis of IBD and the development of cardiometabolic disorders. These dietary patterns contribute to gut microbiota dysbiosis, increased intestinal permeability and chronic low-grade systemic inflammation, while promoting insulin resistance, dyslipidemia and metabolic syndrome. The Western lifestyle thus represents a critical link between gut inflammation and the cardiovascular-endocrine axis (2–4). Emerging evidence suggests that diet-induced alterations in the gut microbiota drive the production of bioactive metabolites that act as key mediators linking intestinal inflammation to vascular pathology. One prominent example is trimethylamine N-oxide, derived from dietary choline and carnitine via microbial metabolism, which promotes atherogenesis by modulating cholesterol handling, enhancing foam cell formation, activating platelets, and impairing endothelial function (5, 6). In parallel, Western diet–associated disruptions in bile acid metabolism lead to the accumulation of secondary bile acids that function as signaling molecules through receptors such as the farnesoid X receptor, thereby influencing lipid metabolism, inflammatory pathways, and vascular homeostasis (3, 7, 8). Moreover, dietary patterns associated with IBD foster intestinal dysbiosis and increased epithelial permeability, facilitating the translocation of microbial products into the systemic circulation. This promotes low-grade endotoxemia, sustaining chronic cytokine production and systemic inflammation (9, 10).

The etiopathogenesis of IBD results from a complex interplay between genetic predisposition, environmental factors (including infections, diet, smoking, medications, stress and socioeconomic status) and the gut microbiota (11). In addition to gastrointestinal manifestations, IBD is increasingly recognized as a systemic disease, with approximately 40% of patients developing extraintestinal complications, including an increased risk of cardiovascular disease. Importantly, IBD should not be considered solely as a localized inflammatory disease of the gastrointestinal tract, but rather as a condition associated with systemic metabolic and endocrine dysregulation (12–14). Adipose tissue, particularly visceral fat, acts as an active endocrine organ, secreting adipokines and proinflammatory cytokines that modulate immune responses and metabolic homeostasis. In IBD, these regulatory pathways are often disrupted, contributing to persistent inflammation with significant endocrine consequences. This metabolic-inflammatory interplay provides a mechanistic framework linking IBD to lipid abnormalities - including the lipid paradox, characterized by reduced circulating lipid levels despite increased cardiovascular risk due to inflammation-related alterations in lipid metabolism leading to lipoprotein dysfunction - as well as increased cardiovascular risk.

Several large cohort studies and meta−analyses have consistently demonstrated an increased risk of atherosclerotic cardiovascular disease (ASCVD) in patients with IBD. Reported relative risk ratios typically range from 1.1 to 1.3 compared with the general population (15–20). The risk is particularly pronounced in younger patients and during periods of active disease or flare−ups, when systemic inflammation is highest (15, 16, 19, 21). This increased cardiovascular risk is not fully explained by traditional factors such as hypertension, diabetes, hypercholesterolemia, or smoking. Instead, it is believed to result from chronic systemic inflammation, alterations in the intestinal microbiota, pro−atherogenic lipid changes, and adverse effects of certain medications, particularly corticosteroids (16, 17, 21–24). Disease activity plays a central role: both clinical and histological inflammation are associated with a higher rate of major adverse cardiovascular events (MACE), while cardiovascular risk approaches baseline during remission (15, 19).

## 2 Possible mechanisms linking IBD with ASCVD

A number of chronically altered physiological processes in patients with IBD may contribute to the development of ASCVD. These include local and systemic inflammation, endothelial dysfunction, gut microbiome abnormalities, hypercoagulable state with increased thrombotic risk, dyslipidemia, adverse effects associated with certain IBD therapies, and nutritional deficiencies (15, 16, 25).

### 2.1 Chronic local and systemic inflammation

Chronic inflammation and inflammatory cytokines, which play a central role in the development of IBD, are also key contributors to the pathogenesis of atherosclerosis and the accelerated development of ASCVD (26, 27). Pro−inflammatory cytokines such as interleukin (IL)−1, IL−2, IL−6, IL−12, IL−17, IL−23, and tumor necrosis factor−alpha (TNF−α), produced by monocytes, epithelial cells, macrophages, endothelial cells, fibroblasts, and dendritic cells, collectively drive the immune response observed in IBD (15, 28). These same cytokines are also implicated in enhanced atherogenesis and an increased risk of ASCVD (25, 29, 30). TNF-α and IL-1β are key initiators of endothelial activation, promoting leukocyte adhesion and vascular inflammation through increased expression of adhesion molecules (31, 32). IL-6 further propagates this response by enhancing acute-phase signaling, endothelial activation, and monocyte recruitment (32). Adaptive immune pathways also contribute, as IL-12 and IL-23 drive Th1 and Th17 responses, increasing IFN-γ and IL-17 production, which promote macrophage activation, oxidative stress, and plaque progression (33–35). Although IL-17 plays a proinflammatory role in the vasculature, its effects may vary depending on the inflammatory milieu (33). In IBD, these mechanisms are amplified by gut barrier dysfunction, which permits translocation of microbial products into the circulation and activates vascular and immune cells (36–38). Importantly, cytokine signaling occurs within an interconnected network: TNF-α and IL-1β augment IL-6 signaling, IL-6 promotes Th17 differentiation, and IL-17 synergizes with TNF-α to sustain vascular inflammation (32–34). Together with oxidative stress and lipid oxidation, these processes link systemic inflammation to atherogenesis. Chronic exposure to this inflammatory milieu in IBD contributes to impaired endothelial repair and increased cardiovascular risk.

In IBD, disruption of the intestinal mucosal barrier facilitates the translocation of microbial lipopolysaccharides (LPS), which in turn stimulate the production of proinflammatory mediators and promote oxidative modification of low−density lipoproteins (LDL) through Toll−like receptor (TLR) signaling. These processes contribute to endothelial injury and the development of atherosclerosis. TLRs play a key role in the pathogenesis of both IBD and atherosclerosis and have been identified within atherosclerotic plaques, where LPS−induced inflammatory activation may also contribute to plaque instability (39, 40). In patients with IBD, the expression of TLR2 and TLR4 is significantly upregulated in dendritic cells compared with healthy controls, thereby amplifying the inflammatory response. TLR−mediated signaling pathways are key drivers of inflammation during atherogenesis. In the setting of elevated cholesterol, vascular dendritic cell subsets contribute to the initiation and propagation of TLR2/TLR4−mediated vascular inflammation (16, 28). Interactions between LPS and TLR4 within the cardiovascular system activate several prothrombotic mechanisms. Endothelial cells respond to TLR4 stimulation by upregulating tissue factor expression while simultaneously downregulating endogenous anticoagulant pathways. TLR4 signaling also promotes the release of von Willebrand factor and factor VIII from endothelial cells, providing essential cofactors for platelet adhesion and thrombus formation. In addition, LPS−activated endothelial cells increase the production of plasminogen activator inhibitor−1 and thrombin−activatable fibrinolysis inhibitor, leading to impaired fibrinolysis and enhanced thrombus stability (41). Platelets themselves express TLR4 and react to LPS exposure with increased activation and aggregation, a phenomenon documented in patients with myocardial infarction (42).

Both innate and adaptive inflammatory cells are involved in the link between IBD and atherogenesis (30). Patients with IBD are characterized by activated T cells, which are also important players in the pathogenesis of atherosclerosis. Activated T cells have been observed within atherosclerotic lesions, similar to findings in CD (25, 28). T cells infiltrating atherosclerotic plaques display a Th1 phenotype, leading to macrophage activation and increased production of pro−inflammatory cytokines such as interferon gamma (IFN−γ). IFN−γ reduces collagen synthesis, rendering the fibrous cap of atherosclerotic plaques more vulnerable to rupture (43).

Macrophages also participate in the formation and progression of atherosclerotic plaques. Retention of LDL particles in the vessel wall by modified proteoglycans initiates plaque development, while oxidation of LDL triggers monocyte recruitment (44). Recruited monocytes differentiate into macrophages and engulf oxidized LDL, forming foam cells. Foam cells secrete pro-inflammatory mediators including IL-1, TNF-α, reactive oxygen species, and other signaling molecules that further amplify vascular inflammation (28). Compared with healthy individuals, patients with IBD exhibit an increased abundance of CD14+ macrophages, which mount an exaggerated immune response against commensal gut microbiota and produce elevated levels of pro−inflammatory cytokines. Similar to IBD, immunocytochemical studies have shown that CD14+ macrophages predominate in human atherosclerotic plaques (45). Moreover, CD14 polymorphisms have been identified as an independent risk factor for myocardial infarction (46).

Neutrophils also contribute to the acceleration of atherosclerosis in patients with IBD (47). It is well established that chronically elevated leukocyte and neutrophil counts are associated with increased cardiovascular risk (48). Neutrophil−driven atherogenesis has been demonstrated in CD, where patients with peripheral atherosclerotic disease show increased expression of calprotectin−related proteins and CXCR2, a cytokine receptor that promotes neutrophil activation (49). Inflammatory signaling also triggers the activation of inflammasomes, particularly the NLRP3 inflammasome, which has been strongly associated with cardiovascular disease. There is a large body of evidence linking NLRP3 activation to arterial thrombosis. The NLRP3 inflammasome is activated by a variety of stimuli - including adaptive immune cells, hypoxia, extracellular ATP, oxidized LDL, reactive oxygen species, and calcium crystals - and its activation promotes atherosclerosis and the formation of necrotic cores in plaques (50). Among the proposed mechanisms, NLRP3 activation induces the release of neutrophil extracellular traps, which increase thrombin generation and platelet activation and promote arterial thrombosis (16, 51). Understanding the shared inflammatory pathways and cytokines involved in both conditions may help identify biomarkers for early detection of disease progression and enable the development of personalized therapeutic strategies aimed at reducing atherosclerosis risk in patients with IBD (28).

### 2.2 Endothelial dysfunction

The endothelium regulates blood flow by preventing platelet and leukocyte adhesion and aggregation, and by limiting permeability to plasma components. Endothelial cells become activated in response to inflammatory mediators and cytokines, leading to increased expression of cell adhesion molecules. In atherosclerosis, endothelial cells express adhesion molecules such as monocyte chemoattractant protein−1 (MCP−1), IL−8, intercellular adhesion molecule−1 and −2 (ICAM−1, ICAM−2), vascular cell adhesion molecule−1 (VCAM−1), E−selectin, and P−selectin. These molecules enhance the expression of receptors that trap monocytes and leukocytes, promoting their recruitment into the subendothelial space and contributing to atherosclerotic plaque formation (52). Increased endothelial permeability, along with structural changes in extracellular matrix macromolecules, facilitates infiltration and retention of cholesterol−rich LDL particles within the arterial wall (53).

In IBD, disruption of the intestinal epithelial barrier permits luminal microbes to penetrate the lamina propria. In response, immune cells secrete proinflammatory cytokines and chemokines, which are well−established mediators of endothelial dysfunction. Human intestinal microvascular endothelial cells isolated from chronically inflamed CD and UC tissue demonstrate enhanced leukocyte adhesion following stimulation with IL−1 or LPS, supporting the role of inflammatory cytokine–mediated endothelial injury in IBD (54, 55). Patients with IBD also exhibit elevated levels of vascular endothelial growth factor (VEGF), a potent angiogenic cytokine that increases vascular permeability (56). Additional inflammatory mediators central to IBD pathogenesis, such as TNF−α, can induce endothelial alterations and impair vascular function by reducing nitric oxide bioavailability (30, 40). TNF−α also enhances interactions between endothelial cells and circulating leukocytes by upregulating adhesion molecules, including VCAM−1 and ICAM−1. Notably, expression of ICAM−1 and VCAM−1 correlates with IBD activity, as their serum concentrations decrease following effective treatment (17, 30). Interleukin−6 (IL−6), another cytokine elevated in IBD, has been also linked to endothelial dysfunction, early atherosclerosis, and ischemic heart disease (40, 57). Inflammatory cytokines, particularly TNF-α and IL-6, also serve as key regulators of the prothrombotic state characteristic of IBD (16, 58).

These prothrombotic mediators activate endothelial cells, driving expression of tissue factor, the primary initiator of the coagulation cascade, while concurrently suppressing natural anticoagulant mechanisms such as the protein C and antithrombin pathways (59). Platelet activation, which is heightened in both number and reactivity in IBD, further amplifies these processes. Activated platelets release CD40 ligand and form platelet-leukocyte aggregates, thereby reinforcing inflammatory signaling and increasing thrombotic potential. These platelet abnormalities correlate with disease activity and contribute to the formation of circulating microparticles expressing tissue factor, providing additional prothrombotic surfaces (16, 60).

### 2.3 Hypercoagulable state

The hypercoagulable state observed in IBD is associated with an increased incidence of both venous and arterial thrombotic events (15, 58). This elevated risk is thought to result from abnormalities in coagulation, fibrinolysis, and platelet function, processes that are partly driven by the effects of pro−inflammatory cytokines (40). In particular, in the active phases of UC and CD, a hypercoagulable and prothrombotic state occurs (61). Elevated levels of coagulation factors - including factor V, factor VIII, fibrinogen, and von Willebrand factor - have been consistently reported in both conditions. Markers of coagulation activation, such as prothrombin fragment 1 and 2, thrombin-antithrombin III complexes, fibrinopeptide A, and fibrinopeptide B, further indicate activation of the coagulation cascade in IBD. In parallel, reduced concentrations of natural anticoagulants - including antithrombin III, protein C, and protein S - underscore the presence of a systemic prothrombotic milieu (62). Whether this spontaneous activation of coagulation pathways represents a primary feature of IBD, a consequence of chronic inflammation, or a process independent of clinical disease activity remains uncertain (28).

Increased platelet activation and aggregation also represent well−established features of IBD and further elevate the risk of ischemic complications. Patients with IBD frequently exhibit platelet abnormalities, including alterations in size, number, and density, compared with healthy individuals. Notably, spontaneous platelet aggregation occurs in more than 30% of patients with IBD, contributing to heightened ischemic risk (63).

Nutritional deficiencies may also contribute to endothelial dysfunction and a procoagulant state. Patients with IBD are at increased risk of vitamin B_12_ and folate deficiencies (64). Deficiencies in these vitamins can lead to hyperhomocysteinemia, which represents an additional risk factor for both endothelial dysfunction and thrombosis. Homocysteine has also been shown to play a role in intestinal inflammation and endothelial injury in IBD. Human intestinal microvascular endothelial cells exposed to homocysteine exhibit features of endothelial activation, including increased VCAM−1 expression, enhanced MCP−1 production, and p38 phosphorylation (65). Furthermore, homocysteine synergizes with TNF−α to amplify endothelial activation, resulting in increased expression of cell adhesion molecules and subsequent T−cell and monocyte adhesion. These mechanisms resemble early steps in the inflammatory cascade that initiates atherosclerosis (28).

### 2.4 Gut microbiome dysbiosis

Both IBD and cardiovascular disease share characteristic alterations in gut microbiota composition (28, 30). In both conditions, microbial diversity is reduced, beneficial *Firmicutes* (particularly butyrate-producing species such as *Faecalibacterium prausnitzii* and *Roseburia hominis*) are depleted, and *Enterobacteriaceae* (especially *Escherichia coli*) and *Streptococcus* species (especially *Streptococcus anginosus* and *Streptococcus dispar*) are enriched (66–70). These changes contribute to decreased butyrate production, loss of epithelial protection, increased apoptosis, and impaired intestinal barrier integrity. IBD-associated dysbiosis is therefore both compositional and functional. Beyond the loss of beneficial anaerobes, an increase in facultative anaerobes (e.g., adherent-invasive *E. coli*, *Proteobacteria*) contributes to enhanced inflammation.

These microbiota changes influence atherosclerosis through multiple mechanisms. Microbial antigens such as LPS translocate into systemic circulation, generating low-grade endotoxemia that activates TLR4, induces inflammatory cascades, and promotes both thrombotic and metabolic disturbances (28, 40). LPS-TLR4 signaling is detected not only in the inflamed gut but also within human atherosclerotic plaques, highlighting a shared inflammatory axis between IBD and vascular disease (39, 71, 72). TLR4 activation increases synthesis of lipid-binding proteoglycans, promoting lipid retention in arterial walls and contributing directly to plaque development (42). Therapeutically, TLR4 represents a potential shared target. Blocking TLR4 can reduce inflammation in colitis models, but impaired mucosal healing limits direct inhibition strategies (73). Interestingly, common ASCVD therapies - including statins, and angiotensin II receptor blockers - exert anti-atherosclerotic effects partly through modulation of TLR4 pathways, suggesting potential overlap in treatment strategies (28). Overall, dysbiosis-induced LPS translocation, TLR4 activation, chronic inflammation, and microbiota-derived metabolites form a mechanistic bridge between IBD and atherosclerosis.

However, other substances produced by bacteria found in IBD may also contribute to vascular damage. Microbial metabolites such as trimethylamine N-oxide (TMAO) - derived from dietary choline and L-carnitine - enhance inflammatory signaling, endothelial activation, and platelet reactivity (5, 6, 74–76). Although TMAO is strongly associated with cardiovascular disease, its role in IBD is more complex: mechanistic studies suggest involvement in autophagy and inflammasome activation, but some clinical studies show lower circulating TMAO levels in IBD patients (77, 78). Other metabolites, including imidazole propionate from histidine metabolism, impair endothelial function and activate proinflammatory PI3K/Akt–FOXO1 pathways (79, 80). In addition, dysbiosis alters bile acid metabolism; when bacteria responsible for bile acid hydrolysis are reduced, lipid absorption increases, promoting atherogenesis (81, 82).

### 2.5 Dysfunctional fatty tissue and obesity paradox

Epidemiological data confirm that patients with IBD have an increased risk of atherosclerotic cardiovascular events, especially during periods of active disease and in the presence of visceral obesity (16, 23, 83). Recently, visceral fat and, to a lesser extent, liver fat have been identified as major determinants of increased carotid atherosclerosis (84). Patients with IBD have significantly higher epicardial fat thickness than the normal population, which may be also associated with an increased risk of subclinical atherosclerosis (85). Visceral, particularly mesenteric, adipose tissue functions as a highly endocrine and immunologically active organ in patients with IBD, which not only locally exacerbates intestinal inflammation but also contributes to increased cardiovascular risk through systemic mechanisms. Dysfunction of mesenteric adipose tissue in IBD is mechanistically linked to atherosclerosis through promotion of chronic inflammation, imbalance of adipokine production, and immune-metabolic dysregulation (86–88). This dysfunctional adipose tissue produces pro-inflammatory adipokines (leptin, resistin, chemerin, visfatin, etc.) that promote local intestinal and systemic inflammation, thereby promoting endothelial dysfunction and atherogenesis (87, 89). Conversely, adiponectin - a cardiovascular protective and anti-inflammatory factor - is produced to a lesser extent in patients with IBD, which correlates with markers of subclinical atherosclerosis, such as carotid intima-media thickness and arterial stiffness (88). Some studies suggest differential adipokine production in patients with CD and UC, which may indicate a different role of mesenteric adipose tissue in different types of IBD (90).

Chronic inflammation in dysfunctional adipose tissue of IBD patients, driven by adipose-derived mediators and gut dysbiosis, leads to persistent activation of monocytes and macrophages, increased production of pro-inflammatory cytokines (e.g., TNF-α, IL-6), and endothelial activation, all of which are central to the development of ASCVD. Additionally, the interplay between gut barrier dysfunction, microbial translocation, and adipose tissue expansion further amplifies systemic inflammation and atherogenic risk (15, 16, 88, 91). In CD particular, hypertrophic mesenteric adipose tissue (“creeping fat”) plays an active role in the pathogenesis of intestinal damage and is increasingly recognized as a contributing factor to atherosclerosis. Mesenteric adipose tissue in CD constitutes a complex immunologically active microenvironment composed of adipocytes, preadipocytes, fibroblasts, mesenchymal stem cells, endothelial cells, and abundant infiltrating immune cells (85, 89). Transcriptomic analyses consistently demonstrate enrichment of immune−related pathways alongside downregulation of genes involved in lipid metabolism and adipogenesis, indicating a shift toward a pro−inflammatory phenotype (92–94). Creeping fat appears to develop predominantly through adipocyte hyperplasia rather than hypertrophy, as evidenced by up to a fourfold increase in adipocyte numbers within affected mesenteric segments. The release of free fatty acids from this metabolically activated tissue further stimulates hyperplasia of smooth muscle cells within the muscularis propria, representing a hallmark of fibrostenotic transformation in CD. The development of creeping fat is closely linked to chronic transmural inflammation and impaired mesenteric lymphatic drainage (95, 96).

Accumulating evidence indicates that mesenteric adipose tissue in CD is not sterile. Multiple studies have identified a distinct microbial signature - primarily composed of *Proteobacteria* - within mesenteric and visceral fat depots, whereas subcutaneous adipose tissue exhibits no comparable colonization (97, 98). The relative abundance of *Proteobacteria* correlates with established biomarkers of disease activity, including fecal calprotectin and serum C−reactive protein (CRP) (99). Moreover, current data suggest that CRP is more than just a biomarker of atherosclerosis and cardiovascular disease and plays an active role in the development of vascular disease (100). Patients in clinical remission tend to exhibit substantially lower levels of pathogenic bacteria within mesenteric fat compared with those with active inflammation. These observations support the concept that bacterial translocation across a compromised intestinal barrier contributes to persistent immune activation within mesenteric adipose tissue, thereby sustaining chronic local inflammation (97, 98, 101).

**Figure 1:**
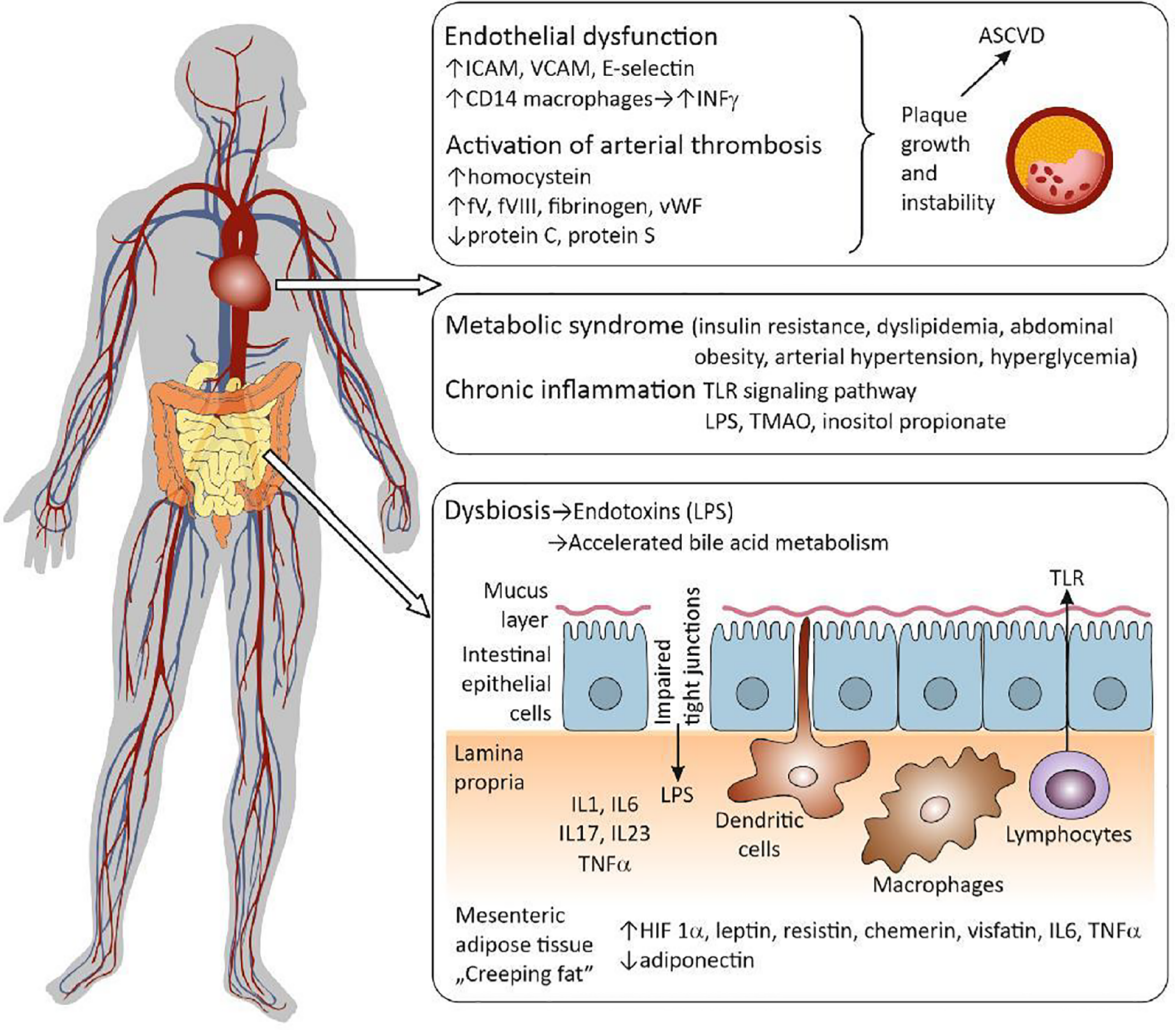
Interaction between the gut, metabolic dysfunction, chronic inflammation, and the cardiovascular system, highlighting mechanisms contributing to ASCVD. Intestinal dysbiosis and impaired gut barrier function facilitate the translocation of bacterial endotoxins - particularly microbial LPS - into the lamina propria of the intestinal mucosa. LPS subsequently activates immune cells, including dendritic cells, macrophages, and lymphocytes, leading to the production of pro inflammatory mediators such as IL 1, IL 6, IL 17, IL 23, and TNF α. Mesenteric adipose tissue, referred to as creeping fat in CD, represents an additional source of inflammatory mediators. This tissue is characterized by increased expression of HIF 1α and dysregulated adipokine secretion, including elevated levels of leptin, resistin, chemerin, and visfatin, along with increased IL 6 and TNF α and reduced adiponectin. Chronic systemic inflammation is driven in part by TLR signaling pathways activated by microbial metabolites such as LPS, TMAO, and inositol propionate. Pro inflammatory cytokines interfere with insulin signaling pathways and promote the development of metabolic syndrome, including insulin resistance, dyslipidemia, abdominal obesity, arterial hypertension, and hyperglycemia. Systemic inflammation further amplifies TLR signaling in endothelial cells, leading to the release of vWF and fVIII, which serve as essential cofactors for platelet adhesion and thrombus formation. Endothelial dysfunction is characterized by increased expression of adhesion molecules, including ICAM, VCAM, and E selectin, as well as activation of CD14 positive macrophages with enhanced IFN γ signaling. This pro inflammatory and pro thrombotic environment promotes arterial thrombosis, reflected by elevated levels of coagulation factors (fV, fVIII, fibrinogen, and vWF) and reduced activity of the anticoagulant proteins C and S. Collectively, these mechanisms contribute to plaque growth, instability, rupture, and the clinical manifestation of ASCVD.Abbreviations: ASCVD, atherosclerotic cardiovascular disease; CD, Crohn’s disease; fV, factor V; fVIII, factor VIII; HIF 1α, hypoxia-inducible factor 1 alpha; ICAM, intercellular adhesion molecule; IBD, inflammatory bowel disease; IFN γ, interferon gamma; IL 1, interleukin 1; IL 6, interleukin 6; IL 17, interleukin 17; IL 23, interleukin 23; LPS, lipopolysaccharide; MAT, mesenteric adipose tissue; TLR, toll like receptor; TMAO, trimethylamine N oxide; TNF α, tumor necrosis factor alpha; VCAM, vascular cell adhesion molecule; vWF, von Willebrand factor.

The expansion of mesenteric adipose tissue supported by intestinal dysbiosis triggers a number of molecular mechanisms that promote chronic inflammatory changes with a systemic impact on the vascular wall. Hypoxia associated with adipocyte expansion activates hypoxia-inducible factor (HIF)-1a (102, 103). HIF-1a can also activate NFkB, thereby promoting the expression of IL-6, TNF-α, IL-1, and MCP-1. Bile acids, lipoproteins (especially oxidized LDL) and a number of other mediators (glutamate, fatty acids, etc.) can also contribute to the activation of NFkB and the expression of pro-inflammatory cytokines (91). This triggers the so-called meta-inflammation, which, together with the hyperactivity of immune cells and systemic metabolic dysfunction associated with insulin resistance, is involved in the development of atherosclerosis. In this way, intestinal dysbiosis, gut adipose tissue expansion, and molecular patterns associated with metabolite damage, along with circulating monocytes, macrophages, and foam cells, contribute to the formation, instability, and rupture of atherosclerotic plaques – see **Figure 1**.

It is important to mention that increased adipose tissue and obesity do not necessarily have a negative impact on IBD. The concept of the “obesity paradox” in IBD reflects the complex and seemingly contradictory influence of elevated body mass index on disease outcomes. Although obesity is traditionally associated with adverse health effects, emerging evidence suggests that it may confer protective benefits in certain contexts, particularly in reducing disease severity and the need for surgical intervention. Several studies indicate that obese patients with IBD may have a lower likelihood of requiring surgery and, in some cases, a less aggressive disease course compared with individuals of normal or low body weight (97, 104, 105). This observation has been partly attributed to increased metabolic reserves provided by adipose tissue during periods of acute illness and physiological stress. However, these findings remain inconsistent, and the overall impact of obesity on disease progression continues to be debated. Obesity has also been associated with IBD risk in a disease-specific manner. Pooled analyses show that obesity is associated with a 34–42% increased risk of developing CD, with evidence suggesting a dose-response relationship between increasing body mass index and disease risk (12). In contrast, prospective data suggest that obesity may be associated with a lower incidence of UC compared with normal-weight individuals. Obesity is also associated with reduced disease remission rates and an increased risk of complicated disease in patients with CD, but not with UC (104).

Despite these potential advantages, obesity is associated with less favorable perioperative outcomes. Obese patients undergoing IBD-related surgery experience higher rates of surgical site infections, delayed wound healing, and increased conversion from minimally invasive to open procedures. In addition, obesity contributes to greater perioperative morbidity, thereby complicating clinical management (12, 87, 105, 106). Furthermore, obesity adversely affects response to pharmacological therapy, particularly biologic agents such as TNF inhibitors (107). Patients with obesity often demonstrate reduced therapeutic efficacy and lower remission rates, likely due to altered pharmacokinetics, including increased drug clearance and a larger volume of distribution leading to subtherapeutic drug levels.

Taken together, the obesity paradox in IBD highlights the dual and context-dependent role of obesity, encompassing both potentially protective and detrimental effects. These observations underscore the need for a balanced and comprehensive approach when evaluating the impact of obesity on disease course, treatment response, and long-term outcomes. Importantly, any potential benefits must be considered alongside the well-established metabolic and cardiovascular risks associated with obesity.

### 2.6 Dyslipidemia

Dyslipidemia is common in IBD, affecting 19-48% of patients, especially men (108). Given the observational and cross-sectional nature of the available evidence, the findings should be interpreted as associative rather than causal, with limited predictive value. In addition, reverse causality and confounding factors (e.g., disease activity, nutritional status) may contribute to the observed relationships between lipid levels and cardiovascular outcomes. A recent meta-analysis including 53 studies showed that patients with IBD had significantly lower levels of total cholesterol (TC), high-density lipoprotein cholesterol (HDL-C), and low-density lipoprotein cholesterol (LDL-C) compared with healthy controls (109). Patients with CD had significantly lower TC concentrations than patients with UC. Disease activity also affected lipid levels: active IBD and moderate-to-severe UC were associated with significantly lower TC and LDL-C levels compared with inactive IBD and mild UC, respectively. The meta-analysis also showed that triglyceride (TG) levels were lower in patients with IBD than in healthy controls; however, the difference did not reach statistical significance. Compared with control groups, the level of TG was significantly lower in CD groups, but not UC groups (110). IBD is therefore associated with a partially atherogenic lipid profile, typically characterized by reduced HDL-cholesterol.

HDL−C levels in patients with IBD show a statistically significant inverse correlation with disease activity and with inflammatory markers such as CRP, TNF, and IL−6 (111, 112). Systemic inflammation alters the structural and functional composition of HDL particles, reducing their cholesterol efflux capacity—the ability to remove cholesterol from the vascular wall. As a result, HDL becomes “dysfunctional,” losing its vasoprotective, antioxidant, and anti−inflammatory properties. Cholesterol ester transfer protein inhibitors (CETPis) were developed to target CETP, a key enzymatic regulator of plasma HDL−C levels. Although these agents substantially increase HDL−C concentrations, clinical trials have not demonstrated a corresponding reduction in cardiovascular risk (111, 112). Interestingly, higher HDL−C levels are positively associated with mucosal healing in patients with IBD. This observation has raised interest in whether therapeutic strategies aimed at modulating HDL-C metabolism might offer clinical benefit in the treatment of IBD. Ongoing research is therefore investigating the potential role of HDL-targeted interventions as adjunctive treatments for IBD (113).

The fluctuating nature of IBD, characterized by cycles of activity and remission, may influence patients’ lipid profiles. Several studies have reported decreases in total cholesterol and LDL−C during clinically active phases of IBD compared with periods of remission, while HDL−C and triglyceride levels appear less affected (110). Conversely, dyslipidemia itself may be associated with more severe IBD, defined by clinical and endoscopic activity as well as elevated high−sensitivity CRP (79). The underlying pathological mechanisms linking chronic inflammation and alterations in lipid metabolism in IBD remain poorly understood. Paradoxically, active inflammation can cause low serum cholesterol levels (hypocholesterolemia), but this condition is often reversible with treatment. Anti-inflammatory drugs can help reduce inflammation, but they can also increase TC and HDL-C levels, often reflecting improved overall health and reduced inflammation (114, 115).

However, some drugs have proatherogenic effects that may be related to changes in the lipid spectrum. Long-term corticosteroid use increases the risk of ASCVD through effects on blood pressure, glycemia, and lipid profiles. Compared with long-term corticosteroid exposure, use of anti-TNF drugs is associated with reduced mortality and a lower risk of MACE in patients with CD (114). Some small molecules (JAK inhibitors, especially tofacitinib) also require caution in patients with pre-existing cardiovascular risk. Induction therapy with both prednisone and tofacitinib significantly increases serum lipid levels in patients with IBD. Relative increases in TC, HDL-C, and LDL-C were significant after treatment with prednisone (+26%, +31%, +12%) and tofacitinib (+20%, +25%, +26%), whereas no changes were observed with other drug classes (115). On the other hand, a meta-analysis including more than 34,000 participants (12,196 with CD and 22,007 with UC) did not find an increased risk of serious adverse cardiovascular events with treatment with small molecule drugs (tofacitinib, upadacitinib) or anti-inflammatory antibodies (infliximab, ustekinumab) compared with placebo during the induction and maintenance phases (116).

Lipid−lowering agents - particularly statins - have been explored for their potential to reduce the risk of IBD and to act as adjunctive therapy due to their anti−inflammatory and microbiota−modulating effects. While experimental models consistently show that statins reduce intestinal inflammation, clinical trials investigating their preventive or disease-modifying effects in humans have yielded mixed and sometimes conflicting results (117). Observational evidence suggests a reduced risk of CD and potentially a milder course of disease in statin users, including fewer hospitalizations and surgeries, although the results for UC are less consistent. However, some studies suggest a modest protective effect also in UC, likely related to their anti-inflammatory effects (118). Recent results from a prospective population-based cohort study - included over 110,000 statin users for primary prevention of cardiovascular disease - documented that statin use was associated with a reduced risk of IBD, with subgroup analyses showing similar reductions in both CD and UC (119). However, the number needed to be treated to prevent one additional case of IBD during 5 years of statin treatment was high (n=2881). Overall, current epidemiological evidence does not yet support statins as a preventive or therapeutic strategy for IBD.

Another recent studies - including Mendelian randomization (MR) analyses - indicate that the impact of lipid−lowering therapies on IBD depends on the specific lipid−regulatory pathway targeted. Genetically predicted inhibition of 3-hydroxy-3-methyl-glutaryl-coenzyme A reductase, the statin target, appears to reduce IBD risk, particularly in CD, whereas genetic variation mimicking inhibition of Niemann-Pick C1-Like 1 protein (NPC1L1) is associated with increased IBD susceptibility (120). In line with this, pharmacologic inhibition of NPC1L1 by ezetimibe - reducing intestinal cholesterol absorption - has been linked to a higher risk of IBD, especially UC (121). Similarly, genetically proxied inhibition of angiopoietin-like protein 3 (e.g., evinacumab) and apolipoprotein C3 (e.g., volanesorsen) may elevate the risk of CD and UC, respectively. Evidence regarding proprotein convertase subtilisin/kexin type 9 (PCSK9) inhibition is mixed: while some analyses show no significant causal effect, others propose that reduced PCSK9 activity may increase IBD risk by altering gut microbial diversity and promoting pro−inflammatory cytokines such as IL−6 and TNF−α (121–123).

Lipoprotein(a) [Lp(a)] represents an additional lipid parameter implicated in heightened cardiovascular risk. Elevated Lp(a) concentrations are recognized as an independent risk factor for ASCVD and acquired aortic valve stenosis, acting through mechanisms such as increased atherogenesis and calcification in the context of chronic inflammation and an increased propensity for arterial thrombosis (124). Lp(a) contributes to inflammatory processes by binding oxidized phospholipids, and inflammatory states—likely mediated by cytokine-driven pathways—can, in turn, stimulate its hepatic production. Although inflammation-induced upregulation of *LPA* gene expression has been observed, the precise molecular mechanisms remain insufficiently defined (125). Recently, MR analysis does not support a causal influence between Lp(a) and immune-mediated inflammatory diseases (126). However, emerging data suggest that individuals with IBD may have increased circulating levels of Lp(a) (127, 128). Supporting this observation, a study conducted in a Chinese cohort reported significantly higher Lp(a) levels in patients with active CD compared with those in clinical remission (129). At present, no approved pharmacological interventions specifically lower Lp(a). Novel therapeutic strategies under investigation include antisense oligonucleotides and small interfering RNA molecules targeting the mRNA encoding apolipoprotein(a), thereby reducing Lp(a) synthesis (124). However, the relevance and therapeutic potential of these agents in patients with IBD remain to be established, and further prospective clinical trials are warranted.

### 2.7 Lipidomics in IBD, its relation to cardiovascular risk

Lipidomics is an emerging branch of systems biology focused on the comprehensive characterization of lipid species within biological systems. Unlike conventional lipid measurements, which are typically limited to total cholesterol and its fractions, lipidomics enables high-resolution profiling of hundreds to thousands of individual lipid molecules, providing deeper insight into metabolic and inflammatory processes. Alterations in the lipidome have been implicated in a wide range of diseases, including metabolic, cardiovascular, and immune-mediated disorders, with growing relevance for cardiovascular risk prediction and mechanistic research (130–132). In IBD, these approaches have gained increasing attention.

Patients with IBD exhibit a characteristic dysregulation of lipid metabolism, which contributes to systemic inflammation, endothelial dysfunction, and increased cardiovascular risk. This phenomenon, often referred to as the “lipid paradox,” reflects the coexistence of reduced circulating cholesterol levels with an elevated incidence of cardiovascular complications. Lipidomic analyses provide a more detailed characterization of these alterations, consistently identifying changes in ceramides, sphingolipids, and phospholipids. These lipid classes not only correlate with IBD activity but are also established predictors of ASCVD, suggesting overlapping pathogenic mechanisms between intestinal inflammation and vascular disease, where inflammatory pathways play a central role in atherogenesis (130, 133).

Recent MR studies further support a potential causal role of specific lipid species in IBD pathogenesis. Distinct lipidomic signatures appear to be associated with IBD subtypes, with phosphatidylcholines and lysophosphatidylcholines more strongly linked to UC, whereas cholesterol esters, diacylglycerols, and lysophosphatidylethanolamines show stronger associations with CD (134). These findings indicate subtype-specific lipid involvement and highlight the complexity of lipid-mediated pathways in IBD. Emerging evidence also suggests that the relationship between the lipidome and IBD may be partially mediated by inflammatory protein networks. Selected lipid classes, including sterol esters, phosphatidylcholines, and sphingomyelins, have been associated with altered levels of circulating immune mediators such as CCL4 and CD6, pointing to a mechanistic link between lipid metabolism and immune activation (135). However, these associations require further validation and should be interpreted with caution.

Although several lipidomic studies have reported altered sphingolipid and phospholipid metabolism in IBD, direct comparisons between CD and UC remain limited and should be interpreted with caution, because lipidomic signatures differ according to biological matrix, disease activity, treatment exposure, age group and analytical platform. In serum NMR-based profiling, both CD and UC showed a distinct metabolic and lipoprotein profile compared with healthy controls, whereas the separation between CD and UC was less pronounced than the overall distinction between IBD and controls (136). Among sphingolipids, lactosylceramide LacCer(d18:1/16:0) has been proposed as a promising CD-associated marker in pediatric serum studies, although subsequent validation data suggest that it may also represent a broader IBD-associated blood marker rather than a universally subtype-specific discriminator (137, 138). By contrast, mucosal lipidomic studies in UC have shown prominent alterations in phosphatidylcholines, ceramides and sphingomyelins, including Cer(d18:1/24:0) and Cer(d18:1/24:2) in treatment-naïve active UC (139). Importantly, these patterns appear to be dynamic: in treatment-refractory UC, several ceramides, sphingomyelin and glycerophospholipid species are decreased compared with treatment-naïve active UC, suggesting lipid remodeling during chronic or treatment-resistant inflammation rather than a fixed UC-specific lipid profile (140). Lysophosphatidylcholine species are generally reduced in active or severe IBD and correlate with inflammatory and lipid parameters, but current evidence does not support their use as robust CD versus UC discriminators (141). Overall, available lipidomic data suggest that CD and UC may differ in selected sphingolipid and phospholipid signatures, but these differences are highly context-dependent and require stratification by disease activity, treatment status and sample matrix before they can be interpreted diagnostically or mechanistically.

Although lipidomics primarily focuses on complex lipid species, related metabolic pathways - particularly those involving short-chain fatty acids (SCFAs) - may also contribute to immune regulation in IBD. SCFAs are products of microbial fermentation of dietary fiber and play an important role in maintaining intestinal homeostasis. Experimental studies demonstrate that SCFAs modulate immune responses through multiple mechanisms, including histone deacetylase inhibition and regulation of macrophage and T-cell function (142–146). Reduced availability of SCFAs, especially butyrate, has been associated with impaired epithelial barrier function and enhanced mucosal inflammation, linking gut microbiota, metabolism, and immune responses (147–150). However, their immunological effects appear to be context- and concentration-dependent (142, 143).

Beyond their diagnostic and mechanistic relevance, lipidomic approaches may improve cardiovascular risk stratification in IBD. Biomarkers such as ceramides and specific fatty acid species have demonstrated promising predictive value compared to traditional lipid parameters, including LDL-cholesterol (130–132). Incorporation of lipidomic profiles into clinical models may therefore enhance individualized cardiovascular risk assessment and support more precise clinical decision-making in patients with IBD.

### 2.8 Other cardiovascular risk factors associated with IBD

Previous studies have shown that patients with IBD do not exhibit a higher incidence of traditional cardiovascular risk factors - such as elevated plasma lipids, glucose levels, altered body composition, or increased blood pressure - compared with the general population (151, 152). However, more recent evidence indicates that, in parallel with the rising prevalence of obesity in the overall population, the incidence of metabolic syndrome (MetS) is also increasing among individuals with IBD. In a cohort of 100,890 U.S. patients with IBD, MetS was present in 34.4% overall (32.4% in UC and 34.3% in CD) (153). MetS clustered with more extensive and severe IBD phenotypes, including pancolitis, acute severe UC, stricturing CD, and a history of CD-related surgery. It was also associated with increased systemic steroid exposure and higher rates of surgery or colectomy, although the risks for strictures and fistulas were comparable between patients with and without MetS. Recently, MR genetic analysis has further demonstrated a causal relationship between genetically predisposed MetS and the development of CD, as well as between genetically predisposed hypertension and UC (154). These findings underscore the need for careful clinical monitoring of IBD patients. Likewise, given the established cardiovascular risks associated with MetS, vigilant cardiovascular risk assessment is essential in patients with IBD.

## 3 Discussion

Evidence from intervention studies specifically addressing optimal ASCVD prevention in patients with IBD remains limited. Current guidelines from both the American College of Cardiology/American Heart Association and the European Society of Cardiology acknowledge chronic inflammatory diseases, including IBD, as independent contributors to ASCVD risk (155, 156). They recommend considering these conditions as risk−enhancing or risk−modifying factors that strengthen the rationale for implementing cardiovascular preventive therapies in patients with borderline or intermediate cardiovascular risk. The cumulative inflammatory burden and the recent activity of inflammation are key determinants of this risk−enhancing effect. In addition to optimal anti−inflammatory therapy, cardiovascular risk in patients with chronic inflammatory conditions should be managed using approaches applied to the general high−risk population. Evidence indicates that traditional risk−reduction strategies - such as lipid−lowering therapy - are equally effective in mitigating the risk of ASCVD in these patients (156).

All individuals with IBD should undergo systematic evaluation for established cardiovascular risk factors, including tobacco use, hypertension, diabetes mellitus, and dyslipidemia, with initiation of appropriate evidence−based therapies as indicated. Current evidence, however, does not support routine use of cardiovascular medications for primary prevention in IBD in the absence of standard indications (16, 20, 22). Lifestyle interventions - such as dietary precautions, stop smoking, regular physical activity, and structured approaches to stress and anxiety management - should likewise be strongly encouraged.

Routine cardiovascular risk assessment and close monitoring are warranted for patients with IBD, particularly during disease flares and hospitalizations (16, 21, 22). Nevertheless, certain pathophysiological mechanisms through which IBD augments ASCVD risk may not be fully captured by conventional clinical risk calculators, potentially resulting in an underestimation of true cardiovascular risk in this population (15). Scores that incorporate markers of inflammatory activity - such as high−sensitivity C−reactive protein - may be more appropriate in these patients than traditional tools like the AHA 10−year ASCVD risk calculator or the European SCORE 2 system (155, 156). Instruments such as the Reynolds Risk Score and the QRESEARCH risk estimator version 3 (QRISK3) integrate inflammatory parameters and therefore offer potential advantages (22, 151). Although QRISK3 was originally designed for individuals with rheumatoid arthritis or systemic lupus erythematosus, it is also considered a suitable tool for estimating cardiovascular risk in patients with IBD (157).

Several noninvasive assessments of subclinical atherosclerosis - including pulse wave velocity for arterial stiffness, carotid ultrasonography for intima-media thickness and atherosclerotic plaque detection, and coronary artery calcium scoring by computed tomography - can further refine cardiovascular risk stratification (16, 151). Nevertheless, data on the use and predictive value of these modalities specifically in the IBD population remain limited. The first study to use carotid ultrasound with atherosclerotic plaque detection to refine cardiovascular risk stratification - allowing reclassification of patients into a very−high−risk category - demonstrated that carotid artery disease was significantly more common in individuals with IBD than in controls (158). In contrast, data from the prospective CLARIFY registry, which evaluated coronary artery calcium (CAC), found no significant differences in median CAC scores between patients with IBD and age− and sex−matched controls, nor between UC and CD subgroups (159). However, among symptomatic patients younger than 60 years, a substantial proportion of obstructive coronary artery disease occurred despite the absence of CAC. This finding was associated with higher risks of myocardial infarction and all-cause mortality and may be particularly relevant for the typically younger IBD population (25). Other, less commonly used markers of subclinical atherosclerosis and endothelial dysfunction - such as carotid intima−media thickness, flow−mediated dilation, and carotid−femoral pulse−wave velocity - have also been reported to show more frequent abnormalities in patients with IBD compared with controls (160).

### 3.1 Cardiovascular medication in IBD patients

#### 3.1.1 Statins

Statins are key agents in reducing cardiovascular risk in patients with chronic inflammatory diseases, both through their lipid-lowering and anti-inflammatory effects. Beyond LDL reduction, they modulate inflammation, improve endothelial function, and reduce cytokine production. They have been shown to lower CRP levels and reduce thrombus formation, suggesting a possible role in attenuating the vascular risk associated with inflammation. Statins are considered safe in IBD and offer benefits beyond lipid-lowering: their anti-inflammatory and immunomodulatory properties may contribute to reducing disease activity and the risk of developing IBD (117–119). Experimental IBD models demonstrate that statins can modulate TNF−α expression in intestinal epithelial cells. By inhibiting the mevalonate pathway, they downregulate NF−κB activation and the production of pro−inflammatory cytokines, suppress Th1/Th17 responses, and enhance regulatory T−cell activity (89). Statins also strengthen epithelial tight junctions, attenuate oxidative stress, and may beneficially influence gut microbiota composition, contributing to broader mucosal protective effects (117).

On the other hand, the use of statins in patients with in IBD may be associated with some concerns, and a balance needs to be struck between the potential anti-inflammatory benefits and unresolved safety concerns. Observational data suggest a reduced risk of CD in statin users, although no consistent association has been observed for UC, highlighting possible disease-specific effects and residual confounding factors (161). The evidence linking statins to microscopic colitis is inconsistent. While earlier meta-analyses reported an increased risk, more recent population-based studies suggest minimal or no clinically relevant risk, suggesting that previously observed associations may reflect bias rather than causality (162). In advanced IBD, statins appear to improve clinical outcomes, with large cohort studies demonstrating reduced risks of surgery, hospitalization, and disease flares, particularly in UC (120, 163). Emerging data further support a potential antifibrotic role in CD, with an approximately 30% reduction in stricture formation in statin users (164). In addition, a growing body of evidence supports a chemopreventive effect against IBD-associated colorectal cancer, with meta-analyses reporting a risk reduction of up to one-third (165). Overall, although statins may confer clinically relevant benefits in IBD, the preponderance of observational evidence and conflicting safety signals highlights the need for prospective studies to clarify their therapeutic role.

Statins are effective in improving lipid levels, reducing inflammatory markers, and - based on many studies - reducing the risk of cardiovascular events. Long-term data consistently demonstrate reductions in arterial stiffness and blood pressure in inflammatory joint diseases (166). In systemic lupus erythematosus, atorvastatin has been shown to slow the progression of coronary artery calcification and improve myocardial perfusion. Other studies have reported improvements in lipid profiles and inflammatory markers, although short follow-up limited the ability to detect reductions in clinical cardiovascular events (167). Evidence in IBD remains limited, but the known CV protective effects of statins support their use in this population. Statins should be an integral part of dyslipidemia treatment, especially in patients taking JAK inhibitors or in patients with longer disease duration, given their ability to reduce cardiovascular risk related to inflammation (168). When LDL targets are unmet, adjunctive agents (e.g., PCSK9 inhibitors, ezetimibe, bempedoic acid) may be added based on individual risk profiles (166).

#### 3.1.2 Aspirin

Aspirin use for primary and secondary cardiovascular prevention in chronic inflammatory diseases such as IBD generally follows recommendations for the general population. Current guidelines recommend low-dose aspirin (75–100 mg/day) for secondary prevention in individuals with established ASCVD. In primary prevention, routine aspirin use is generally not recommended, with recommendations focusing on shared decision-making in adults aged 40–59 years at high cardiovascular risk (155, 156). Despite its shared gastrointestinal toxicity profile with other NSAIDs, low−dose aspirin has not been linked to higher incidence or exacerbation of IBD (151). This may reflect its weaker COX−2 inhibition at low doses and its potential direct mucosal protective and anti−inflammatory effects. The use of low-dose aspirin for primary or secondary cardiovascular prophylaxis does not appear to be associated with an increased risk of disease exacerbations (25).

#### 3.1.3 Antihypertensive drugs

IBD patients often receive various antihypertensive agents. There is currently no evidence supporting the use of any specific antihypertensive class uniquely for patients with IBD. Therefore, ACE inhibitors and angiotensin II receptor blockers - the standard first-line drugs for the treatment of hypertension in the general population - should also be considered as first-line drugs in patients with IBD, unless contraindications or individual clinical factors indicate otherwise (25). Whether specific drug classes influence IBD activity or cardiovascular risk remains unclear. Components of the renin–angiotensin system are implicated in intestinal inflammation and fibrosis, and use of ACE inhibitors or sartans may be associated with improved outcomes (151). Telmisartan, in particular, has shown anti−inflammatory, antioxidant, and anti−apoptotic effects in murine models of IBD (169). In contrast, one small retrospective study reported a higher relapse rate in IBD patients using β−blockers, although more research is needed to clarify these potential associations (170).

#### 3.1.4 GLP-1 receptor agonists

GLP−1 receptor agonists (GLP-1 RA) have recently attracted interest for their potential role in IBD and CV disease. A recent population−based cohort study involving 10,855 patients with immune−mediated inflammatory diseases (including 1,562 with IBD) and type 2 diabetes reported that treatment with GLP-1 RA was associated with a reduced risk of all−cause mortality and MACE compared with a CV neutral active comparator (171). Preclinical data suggest GLP-1 RA reduce systemic inflammation through weight−loss mediated effects and direct modulation of gut immune pathways, including altering intraepithelial lymphocyte activity (89). Mouse studies indicate upregulation of IL−33, mucin 5B, and CCL20, molecules typically reduced in IBD, as well as enhanced expression of tight−junction related genes, suggesting improved barrier integrity and reduced bacterial translocation (172). Meta−analytic evidence across various diseases shows reductions in TNF−α and CRP with GLP−1 RA (173). Observational human studies show heterogeneous but generally favorable findings: some report reduced flare risk or IBD course, others show neutral effects aside from fewer hospitalizations. Importantly, none demonstrated harm. However, most studies are limited by small sample size, varying methodologies, and confounding factors such as concurrent therapy, baseline disease activity, and obesity−related comorbidities. The most consistent conclusion is that GLP−1 RA do not worsen IBD (7). Emerging evidence from a retrospective cohort study further suggests that semaglutide is effective for sustained weight loss in individuals with obesity and IBD, without increasing IBD−related complications, such as the need for corticosteroids or hospitalization (174). These findings support the safety and potential utility of GLP-1 receptor agonists as a weight management option in patients with IBD, particularly in those with additional features of MetS.

### 3.2 Anti-inflammatory medications and CV risk

Disease activity is a major determinant of cardiovascular risk in IBD. Both histological and clinical inflammation are associated with higher rates of MACE, with the risk returning to baseline during remission (15, 19). Effective control of intestinal inflammation, judicious use of corticosteroids, and adequate treatment of traditional cardiovascular risk factors are recommended as the best prevention of ASCVD (16, 20, 22).

#### 3.2.1 Biologic agents, monoclonal antibodies

Among IBD therapies, anti-TNF biologics (infliximab, adalimumab, golimumab) have the strongest evidence for reducing cardiovascular risk. Observational studies and meta-analyses consistently demonstrate decreased rates of acute arterial events - including coronary artery disease and cerebrovascular events - likely through suppression of systemic inflammation and improved disease control (15, 16, 175). Comparative analyses suggest that anti-TNF agents may confer greater protection against MACE than other biologic classes (175, 176). However, anti-TNF therapy is not without cardiovascular considerations. These agents may exacerbate pre-existing moderate to severe heart failure and have been associated with new arrhythmias in susceptible patients. Careful selection is advised in individuals with underlying cardiac dysfunction (176).

Non−TNF biologics generally exhibit excellent cardiovascular safety profiles. Vedolizumab and ustekinumab have not been associated with increased MACE, hypertension, or dyslipidemia, and appear CV neutral in prospective studies (175, 176). IL−23 inhibitors such as risankizumab and guselkumab likewise demonstrate low rates of cardiovascular events, although rare reports of arrhythmias (e.g., atrial fibrillation) have emerged and require further study (176).

#### 3.2.2 Janus kinase inhibitors, sphingosine 1 phosphate receptor modulators

Janus kinase (JAK) inhibitors (tofacitinib, upadacitinib) and sphingosine-1-phosphate (S1P) receptor modulators (ozanimod, etrasimod) are small molecular drugs approved for the treatment of IBD, whose use in clinical practice may be limited due to cardiovascular issues. JAK inhibitors are most often associated with adverse changes in cardiovascular risk factors. According to the international Delphi consensus, the available evidence does not demonstrate a higher risk of cardiovascular events with JAK inhibitors in the overall IBD population, although it might be increased in patients with an unfavorable cardiovascular profile (177). They increase TC, LDL-C and HDL-C, with the greatest impact seen in patients with baseline cardiovascular risk (115, 151). JAK inhibitors also carry warnings regarding venous thromboembolism and, in some populations, an increased risk of MACE. While the absolute risk in IBD appears to be low, increased vigilance is warranted in high-risk individuals (22, 178, 179).

S1P receptor modulators (most notably ozanimod) may be associated with transient bradycardia and atrioventricular conduction abnormalities, particularly after the first dose. These effects necessitate monitoring, especially in patients with known conduction disease. S1P receptor modulators may be associated with new-onset hypertension (178).

#### 3.2.3 Corticosteroids

Adverse cardiovascular effects of corticosteroids are particularly evident with long-term use. These include insulin resistance, central obesity, hypertension, dyslipidemia, and hyperglycemia (25, 151). Corticosteroids have been shown to increase the risk of ischemic heart disease in patients with IBD, but the data are rather inconsistent (16, 25, 40, 151). The increased risk of MACE and cardiovascular mortality with long-term steroid use was particularly evident compared with anti-TNF therapy use (114, 151). It is difficult to distinguish whether the increase in cardiovascular events in the long term is due to the adverse metabolic effects of steroids or to uncontrolled disease activity. Overall, corticosteroids may have beneficial short-term anti-inflammatory effects, and in the long term, gradual withdrawal is appropriate, and as soon as disease activity permits, appropriate transition to combination therapy with another anti-inflammatory drug and, if necessary, complete withdrawal of corticosteroids is recommended (16, 156).

### 3.3 Other anti-inflammatory drugs

Traditional immunomodulators such as azathioprine and methotrexate do not have a consistent effect on the cardiovascular system, and therefore monitoring for hepatic, hematological and malignancy-related toxicities is necessary (151). Cyclosporine may increase the risk of hypertension and dyslipidemia, and tacrolimus may lead to diabetes (22). 5-aminosalicylates (5-ASA) have been found to be associated with a reduction in cardiovascular events (40, 151); however, the data are inconsistent. On the other hand, 5-ASA have been associated with side effects such as myocarditis and pericarditis (25, 151). **Table 1** summarizes the cardiovascular risk and other consequences of IBD treatment.

**Table 1:** Cardiovascular effects of anti-inflammatory therapy.

Anti-inflammatory drugs	Cardiovascular risk and other consequences
Corticosteroids	- Long-term use leads to increased CV risk (hypertension, obesity, hyperlipidemia, insulin resistance, ischemic heart disease - Not recommended for maintenance therapy
5-aminosalicylates	- Low-risk of ischemic heart disease - Associated with pericarditis, myocarditis and arterial stiffness - Protective effect outweighs risks
Thiopurines	- Risk of pericarditis and arrhythmias - No clear CV benefit
TNF-α antagonists (infliximab, adalimumab)	- Improve CV outcomes - Reduce arterial stiffness - Risk of exacerbation of pre-existing heart failure - Associated with new arrhythmias in susceptible patients - Careful selection is advised in individuals with underlying cardiac dysfunction - High-dose of infliximab is contraindicated in NYHA class 3-4
IL 12/23 inhibitors (ustekinumab)	- Low CV risk or neutral influence
IL−23 inhibitors (risankizumab, guselkumab)	- Low CV risk - Rare reports of arrhythmias
Anti-integrins (vedolizumab)	- May improve CV outcomes - Caution of cerebrovascular hemorrhage
JAK inhibitors (tofacitinib, upadacitinib)	- Potential risk of CV disease, venous thromboembolism and dyslipidemia especially in older adults (higher risk in tofacitinib than upadacitinib) - Low absolute CV risk - Increased vigilance is warranted in high-risk individuals
S1P receptor modulators (ozanimod, etrasimod)	- Rare MACE events and arrhythmias: bradycardia, atrial-ventricular block - These effects necessitate monitoring, especially in patients with known conduction disease - Associated with new-onset hypertension in susceptible patients

CV, cardiovascular; MACE, major adverse cardiovascular events; TNF-α, tumor necrosis factor alpha; IL, interleukin; JAK, Janus kinase; S1P, sphingosine-1-phosphate.

## 4 Conclusion

Evidence from a number of large cohort studies and meta-analyses suggests a modest but consistent increased risk of ASCVD in patients with IBD. The excess cardiovascular risk is not fully explained by traditional risk factors and is thought to be due to chronic systemic inflammation, dysbiosis, proatherogenic lipid profiles, and adverse effects of certain medications. Effective control of intestinal inflammation and aggressive modification of traditional risk factors are recommended. In summary, aggressive control of intestinal inflammation remains the most effective strategy for reducing cardiovascular risk in IBD. Among available therapies, anti-TNF biologics have the strongest evidence of cardiovascular benefit. Other biologics, including vedolizumab and ustekinumab, appear to be CV safe, whereas JAK inhibitors and S1P modulators require more cautious use in patients with pre-existing risk factors. Cardiovascular risk should be assessed regularly, especially during flare-ups. Standard risk calculators may underestimate risk in IBD, and tools that incorporate inflammatory markers (e.g., Reynolds risk score, QRISK3) may be more appropriate. Noninvasive measures of subclinical atherosclerosis - such as carotid ultrasonography, pulse wave velocity, and coronary artery calcification score - may refine risk stratification, although data in IBD are limited. Routine primary prevention with cardiovascular medications is not recommended without standard indications, while lifestyle interventions - including smoking cessation, healthy diet, physical activity, and stress management -are strongly recommended. Lifestyle optimization, aggressive treatment of traditional risk factors, and consideration of statin therapy should be part of the management of IBD patients at increased cardiovascular risk. Statins are key agents in reducing cardiovascular risk in patients with chronic inflammatory diseases, both through their hypolipidemic and anti-inflammatory effects. GLP-1 RAs have recently attracted interest for their potential role in the treatment of IBD and cardiovascular disease. Their administration has been associated with a reduced risk of all-cause mortality and MACE. GLP-1 RAs reduce systemic inflammation and positively influence all components of the metabolic syndrome, including visceral obesity. Dysfunctional adipose tissue and abnormal lipid composition therefore become important modifiable factors for the development of cardiovascular complications in IBD, which deserve further investigation.
